# Evaluation of LCN2 and miR-8078 as diagnostic biomarkers for congenital heart disease-associated pulmonary arterial hypertension

**DOI:** 10.1093/eschf/xvag034

**Published:** 2026-02-10

**Authors:** Qianqian Chen, Dongdong Zheng, Wei Zhang, Ying Hua, Wei Wang, Rong Huang, Xiaofei Li

**Affiliations:** Department of Cardiology, Affiliated Hospital of Nantong University, No. 20 Xisi Road, Nantong 226000, Jiangsu Province, China; Department of Critical Care Medicine, Nantong Third People's Hospital, No. 60 Middle Qingnian Road, Nantong 226000, Jiangsu Province, China; Department of Cardiology, Affiliated Hospital of Nantong University, No. 20 Xisi Road, Nantong 226000, Jiangsu Province, China; Department of Cardiology, Affiliated Hospital of Nantong University, No. 20 Xisi Road, Nantong 226000, Jiangsu Province, China; Department of Cardiology, Affiliated Hospital of Nantong University, No. 20 Xisi Road, Nantong 226000, Jiangsu Province, China; Department of Cardiology, Affiliated Hospital of Nantong University, No. 20 Xisi Road, Nantong 226000, Jiangsu Province, China; Department of Cardiology, Affiliated Hospital of Nantong University, No. 20 Xisi Road, Nantong 226000, Jiangsu Province, China; Department of Cardiology, Affiliated Hospital of Nantong University, No. 20 Xisi Road, Nantong 226000, Jiangsu Province, China

**Keywords:** Congenital heart disease, Pulmonary arterial hypertension, Lipocalin 2, microRNA-8078

## Abstract

**Introduction:**

This study aimed to evaluate the diagnostic value of lipocalin 2 (LCN2) and microRNA-8078 (miR-8078) in congenital heart disease-associated pulmonary arterial hypertension (CHD-PAH).

**Methods:**

Seventy-six patients were diagnosed with CHD-PAH according to established clinical guidelines (including mean pulmonary arterial pressure (mPAP), pulmonary artery wedge pressure, and pulmonary vascular resistance) via right heart catheterization. Based on their mPAP, they were stratified into non-PAH (mPAP < 25 mm Hg; *n* = 28), mild PAH (25 ≤ mPAP < 35 mm Hg; *n* = 21), and moderate-to-severe PAH (mPAP ≥ 35 mm Hg; *n* = 27) groups. Plasma LCN2 levels and miR-8078 expression were quantified using Enzyme-linked immunosorbent assay and RT-qPCR, respectively. The diagnostic value was analysed using receiver operating characteristic curves. Correlation analysis assessed associations between biomarkers and hemodynamic parameters. Multi-variate logistic regression identified independent predictors of CHD-PAH.

**Results:**

Plasma LCN2 (135.1 [40.2] vs 67.7 [17.7] ng/ml; *P* < .05) and relative miR-8078 expression (4.2 ± 1.1 vs 2.3 ± 1.3 fold; *P* < .05) were significantly elevated in the moderate-to-severe PAH group compared with the non-PAH group. Both markers showed positive correlations with mPAP (LCN2: *r* = 0.691, *P* < .001; miR-8078: *r* = 0.481, *P* < .001) and pulmonary artery systolic pressure (LCN2: *r* = 0.579, *P* < .001; miR-8078: *r* = 0.391, *P* < .001). Notably, LCN2 levels positively correlated with miR-8078 expression (*r* = 0.407, *P* < .001). For diagnosing moderate-to-severe PAH, the area under the curve (AUC) was 0.883 for LCN2 and 0.749 for miR-8078. The combined model yielded a numerically higher AUC of 0.896, but did not significantly differ from LCN2 alone. Univariate regression analysis identified both LCN2 and miR-8078 as significant predictors of CHD-PAH. LCN2 was identified as an independent risk factor for CHD-PAH.

**Conclusion:**

Plasma LCN2 and miR-8078 are significantly elevated in patients with CHD-PAH and correlate positively with hemodynamic severity. LCN2, in particular, serves as a robust independent biomarker for the diagnosis and severity assessment of CHD-PAH. Consequently, LCN2 and miR-8078 hold promise as potential non-invasive biomarkers for the diagnosis and severity assessment of CHD-PAH.

## Graphical Abstract

**Graphical Abstract:**
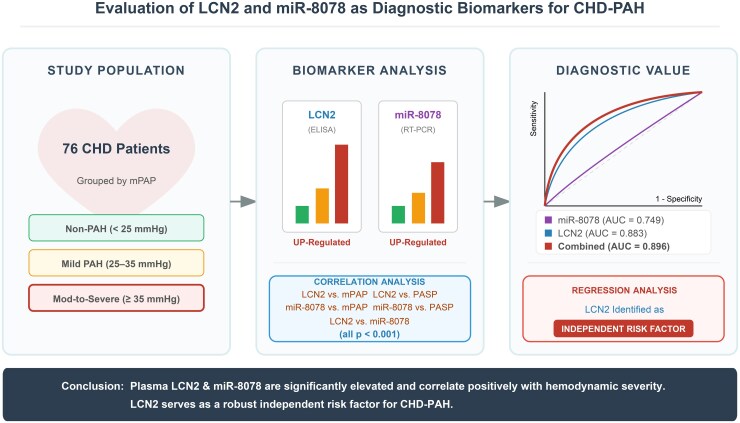

## Introduction

Congenital heart disease is one of the most common birth defects worldwide and represents a serious health problem due to its complications. As a potentially fatal complication of congenital heart disease, pulmonary hypertension has an incidence of ∼10% and results in an increased right ventricular afterload, ultimately leading to heart failure or even death.^1^ Pulmonary arterial hypertension is recognized as the most common type of pulmonary hypertension, and it is known to significantly affect patients’ lifespan.^2^ The aetiology and mechanisms of congenital heart disease-associated pulmonary arterial hypertension (CHD-PAH) are diverse and complex, including vasoconstriction, pulmonary vascular remodelling, thrombosis, hypoxia, inflammation, immune dysfunction, dysregulation of bone morphogenetic protein receptor 2 signalling, impaired RhoA/Rho-kinase inhibitor signalling, and genetic factors.^3^ As there are no obvious clinical signs at an early stage, patients with CHD-PAH are often diagnosed with resistant PAH at a late stage, resulting in a poor prognosis. Therefore, early diagnosis and accurate severity assessment are paramount for guiding therapeutic strategies and improving patient outcomes.

The current gold standard for diagnosis, right heart catheterization (RHC), is invasive, expensive, and not suitable for routine screening or frequent follow-up. Non-invasive methods, such as echocardiographic estimation of pulmonary artery systolic pressure (PASP) from tricuspid regurgitation velocity, are widely used but are susceptible to operator-dependent variability and inaccuracies. Although brain natriuretic peptide (BNP) and N-terminal proBNP (NT-proBNP) are the only biomarkers currently recommended by guidelines for prognostic assessment in PAH, their utility is limited by a lack of specificity for CHD-PAH and their levels can be influenced by confounding factors such as age, gender, and overall cardiac function. Consequently, there is an urgent clinical need for novel, objective, and non-invasive biomarkers for the early detection, severity stratification, and longitudinal monitoring of CHD-PAH.

In the search for such biomarkers, lipocalin-2 (LCN2) has emerged as a promising candidate. LCN2 is a secreted glycoprotein initially discovered in human neutrophil granules, which belongs to the lipocalin superfamily of lipid-binding proteins. It is involved in modulating the progression of chronic renal disease via influencing inflammation, epithelial-mesenchymal transition, and cell activities.^4,5^ LCN2 is known to be highly expressed in lung tissues obtained from the rat model of pulmonary hypertension, and it significantly promotes pulmonary artery smooth muscle cell (PASMC) proliferation by reducing oxidative stress, enhancing intracellular iron levels, and activating the phosphatidylinositol 3-kinase (PI3K)/Akt pathway.^6–8^ These mechanisms and pathways are consistent with the essential causes of the small pulmonary arterial remodelling seen in PAH. Clinical studies have already shown that the increased plasma LCN2 in children with CHD-PAH is significantly positively correlated with invasive hemodynamic indicators, suggesting that it is conducive to assessing and predicting the severity of PAH.^4,6^ Hence, further verifying whether LCN2 has potential as a marker of CHD-PAH is a focus of the current research.

MicroRNA (miRNA) played pivotal role in various cell processes, including cell growth, differentiation, survival, and lipid metabolism.^9^ Furthermore, the abnormal expression of miRNA caused by hypoxia is involved in the development of PAH. In addition, miRNA functions as a regulator of PASMC proliferation and migration.^10^ For instance, miR-371b-5p suppresses pulmonary artery endothelial cell (PAEC) apoptosis in rats with PAH induced by colchicine through the PTEN/PI3K/Akt/eNOS-AP-1 and KLF-2 pathways.^11^ Moreover, miRNA-Let-7b down-regulates ACE2 by directly targeting its coding sequence, thereby inducing PASMC proliferation and migration, which accelerates hypoxia-induced PAH development.^12^ Such findings indicate the close relationship between different miRNAs and PAH, highlighting the significance of these miRNAs as promising indicators of PAH. However, miR-8078 remains largely uninvestigated in this context. Interestingly, miR-8078 has been identified as a core miRNA in the pathogenesis of rheumatoid arthritis (RA),^9^ a disease where the PI3K/AKT signalling pathway plays a critical role. This pathway is also strongly implicated in the pathobiology of PAH. For example, up-regulation of miR-371b-5p has been shown to inhibit apoptosis in PAECs via the PI3K/Akt pathway in a PAH rat model,^11^ and knockdown of miR-1 was found to alleviate right ventricular hypertrophy and fibrosis through mechanisms involving this same pathway.^13^ Based on this evidence, we hypothesized that miR-8078, potentially linked to the PI3K/AKT pathway, could serve as a novel and previously unexplored biomarker for CHD-PAH.

Therefore, this study was designed to investigate the correlation between plasma levels of LCN2, miR-8078, and the severity of PAH in patients with CHD, aiming to evaluate their potential clinical relevance in the diagnosis and assessment of CHD-PAH.

## Methods

### Subjects

A total of 76 CHD patients who underwent RHC at our hospital were enrolled in this study. The present study was carried out with the approval of the Ethics Committee of Nantong University Affiliated Hospital (2016-52), and all the subjects signed the informed consent form.

The cohort primarily consisted of patients with non-cyanotic CHD (*n* = 72, 94.7%), including 31 cases of atrial septal defect, 25 cases of ventricular septal defect, and 16 cases of patent ductus arteriosus. A smaller subgroup of patients had cyanotic CHD (*n* = 4, 5.3%), all of whom were diagnosed with Tetralogy of Fallot. All patients were undergoing corrective catheter-based interventional therapy.

The diagnosis of CHD-PAH was established according to established guidelines, requiring a complete hemodynamic profile of mean pulmonary arterial pressure (mPAP) ≥25 mm Hg, a pulmonary artery wedge pressure ≤15 mm Hg, and a pulmonary vascular resistance (PVR) ≥3 wood units. For the purpose of evaluating biomarker correlation with disease severity, patients were divided into three groups based on their pulmonary hypertension severity and the Chinese Guidelines for the Diagnosis and Treatment of Pulmonary Hypertension (2021 Edition)^1^: Moderate-severe PAH group (CHD patients with moderate or severe PAH, mPAP ≥35 mm Hg, *n* = 27), Mild PAH group (CHD patients with mild PAH, 25 mm Hg ≤ mPAP <35 mm Hg, *n* = 21), and No PAH group (CHD patients without PAH, mPAP <25 mm Hg, *n* = 28).

The inclusion criteria were as follows: patients aged >18 years who were diagnosed with CHD, with or without PAH. Moreover, patients were excluded if they met any of the following criteria: (i) pulmonary hypertension attributed to causes other than CHD (i.e. WHO PH Groups 2, 3, 4, or 5); (ii) a documented history of atrial fibrillation, other clinically significant arrhythmias, or the presence of an implanted cardiac pacemaker; (iii) prior or current treatment with PAH-specific targeted therapies; (iv) severe hepatic dysfunction, including cirrhosis, chronic hepatitis B/C, or liver transaminases >3 times the upper limit of normal; (v) chronic kidney disease, defined as an estimated glomerular filtration rate <60 ml/min/1.73 m²; (vi) active systemic bacterial or viral infections within 4 weeks of enrolment; (vii) a current diagnosis of malignancy or a history of cancer within the past 5 years; or (viii) other significant comorbidities known to alter LCN2 or miR-8078 levels, such as coronary artery disease, cerebrovascular disease, neurodegenerative, or psychiatric disorders (e.g. Parkinson’s disease, cognitive impairment, depression, anxiety), and autoimmune conditions like RA.

### Collection of clinical data

General information about the participating patients was collected, such as their age, gender, height, weight, and various laboratory indicators, including their complete blood cell count, hepatic and renal function, blood glucose, blood gas analysis, electrocardiography, echocardiography, and chest computed tomography. Key hemodynamic parameters, including mPAP, PVR, and mixed venous oxygen saturation (SvO2), were measured intraoperatively during the procedure.

### Specimen determination

After admission, venous blood was collected from the CHD patients while they were in the fasting state. The blood samples were centrifuged at 3000 rpm for 10 min within 30 min of collection. The harvested supernatant was aliquoted and reserved at −80°C and subjected to a maximum of one freeze-thaw cycle. Enzyme-linked immunosorbent assay (ELISA) and reverse transcription polymerase chain reaction (RT-PCR) were performed to measure the patients’ plasma LCN2 and miR-8078 levels.

### Plasma LCN2 measurement by ELISA

Plasma LCN2 concentrations were quantified using a commercial sandwich ELISA kit (Human Lipocalin-2/NGAL ELISA kit, R&D Systems, USA; Cat. No. QK1757) according to the manufacturer’s instructions. The sensitivity of this assay is 0.033 ng/ml, and the assay range is 0.3–10 ng/ml. Prior to the assay, reagents and samples were brought to room temperature. Plasma samples were diluted 1:20 with the provided diluent. Fifty microliters (50 µl) of standards or diluted samples were added to designated wells of a 96-well plate. Subsequently, 50 µl of an antibody mixture was added to each well (excluding the blank well). The plate was sealed and incubated for 1 h at room temperature on a horizontal orbital microplate shaker (500 ± 50 rpm). After incubation, the plate was washed three times using an automated plate washer with 400 µl of wash buffer per well. Following the final wash, the plate was inverted and tapped on absorbent paper to remove residual liquid. Next, 100 µl of substrate solution was added to each well, and the plate was incubated for 20 min at room temperature in the dark. The reaction was terminated by adding 50 µl of stop solution to each well. The optical density (OD) was measured at 450 nm within 5 min using a Bio-Tek microplate reader (USA). All samples and standards were measured in duplicate, and the mean values were used for statistical analysis. The intra-assay and inter-assay coefficients of variation (CV) were maintained below 10%. A standard curve was generated, and the concentrations of LCN2 in the samples were calculated by interpolating the net OD values and multiplying by the dilution factor of 20.

### Plasma miR-8078 quantification by RT-qPCR

Hemolysis was assessed by visual inspection, and samples showing signs of hemolysis were excluded. Total RNA was extracted from 200 µl of plasma using a Trizol-based method. Briefly, 1 ml of Trizol reagent (Vazyme, Nanjing, China) was added to the plasma sample, mixed thoroughly, and incubated for 5 min at room temperature. After adding 200 µl of chloroform and vigorous shaking, the mixture was incubated on ice for 15 min and then centrifuged at 14 000 rpm for 15 min at 4°C. The upper aqueous phase was transferred to a new tube, and RNA was precipitated by adding an equal volume of isopropanol and 1–2 µl of Glycogen (Biosharp, Beijing, China). The samples were incubated overnight at −80°C. Following centrifugation at approximately 18,600 × g for 15 min at 4°C, the RNA pellet was washed with 300 µl of pre-chilled 75% ethanol. The pellet was then air-dried at room temperature for 5–10 min and dissolved in 10 µl of RNase-free water. RNA concentration and purity were assessed using a spectrophotometer, with an OD260/280 ratio between 1.8 and 2.0 considered acceptable.

First-strand cDNA was synthesized from 1 µg of total RNA using a miRNA First-Strand cDNA Synthesis Kit (poly(A) tailing method; Servicebio, Wuhan, China). The reaction was performed in a 20 µl volume containing 4 µl of 5× Reaction Buffer, 1 µl of miRNA RT Enzyme Mix, and total RNA. The thermal profile was 37°C for 60 min, followed by enzyme inactivation at 85°C for 5 s.

Real-Time Quantitative PCR (RT-qPCR) was performed using PowerUp™ SYBR® Green Master Mix (Vazyme, Nanjing, China) on an Applied Biosystems 7500 Real-Time PCR System. The primers were synthesized by Sangon Biotech (Shanghai, China), with the following sequences: miR-8078 (Forward: 5′-TCTAGGCCCGGTGAGAGACT-3′) and U6 snRNA (Forward: 5′-ATGTCTTTGGTGCTCGCTTC-3′). A universal reverse primer provided by the RT kit was used. The 10 µl reaction mixture included 5 µl of 2× PowerUp™ SYBR® Green Master Mix, 0.5 µl of forward primer (10 µM), 0.5 µl of universal reverse primer (10 µM), 1 µl of cDNA, and 3 µl of nuclease-free water. The thermal cycling conditions were: 95°C for 10 min, followed by 40 cycles of 95°C for 15 s and 60°C for 60 s. The relative expression of miR-8078 was calculated using the 2^−ΔΔCt^ method, with U6 serving as the endogenous control for normalization. To ensure the reliability of U6 as a reference gene in our specific experimental setting, the raw Ct values of U6 were analysed across all samples. No statistically significant difference in U6 expression was observed between the experimental and control groups, confirming its stability as a normalizer in this study. All RT-qPCR assays were performed in triplicate to ensure reproducibility. To minimize variability, all samples were handled on ice.

### Statistical analysis

The statistical analyses in this study were conducted using IBM SPSS Statistics version 26 software. Quantitative data close to or following normal distribution were displayed as the mean ± standard deviation ($\bar{x}\pm s$). Data comparisons between two or more groups were performed with an independent sample *t*-test and a one-way analysis of variance (ANOVA) after testing for the homogeneity of the variances. The median and inter-quartile range (IQR) were used to represent quantitative data with a non-normal distribution, and the differences between groups were analysed using the Mann–Whitney *U* test and the Kruskal–Wallis non-parametric test. Count data were represented by the corresponding numbers or proportions using the χ^2^ test or Fisher’s exact test. Spearman’s rank correlation analysis was applied for the estimation of the correlation among the variables. The independent risk factors for CHD-PAH were identified by means of logistic regression analysis. The receiver operating characteristic (ROC) curve and the area under the curve (AUC) were plotted using MedCalc statistical software version 19.6.3 and GraphPad Prism 9. Statistical significance was set as a *P*  *<* .05.

## Results

### General clinical data analysis

A total of 76 CHD patients who underwent RHC were included in this study. As shown in *Table 1*, there were clear differences among the groups in terms of the patients’ age, New York Heart Association (NYHA) functional class, white blood cell count, red blood cell distribution width, fasting blood glucose level, prothrombin time, D-dimer level, NT-proBNP level, left atrial diameter, peripheral oxygen pressure, mPAP, PASP, and PVR (*P* < .05). In contrast, no noticeable differences were observed among the groups in relation to the patients’ gender distribution, body mass index (BMI), blood pressure, haemoglobin level, transaminase level, renal function, activated partial thromboplastin time, lipid profile, left ventricular systolic and diastolic diameters, pulmonary-to-systemic flow ratio, and mixed venous oxygen saturation (*P* > .05).

**Table 1:** General clinical data

Parameters	No PAH group (*n* = 28)	Mild PAH group (*n* = 21)	Moderate-severe PAH group (*n* = 27)	*P*-value
Female [*n* (%)]	15 (53.6)	16 (76.2)	21 (77.8)	.115
Age (years)	40.9 ± 14.5	46.8 ± 16.1	54.3 ± 16.8	.01
BMI (kg/m²)	23.8 ± 3.7	23.4 ± 3.3	23.7 ± 4.4	.936
Systolic blood pressure (mm Hg)	124.1 ± 17.5	127.9 ± 16.3	130.0 ± 19.3	.472
Diastolic blood pressure (mm Hg)	76.0 ± 12.4	75.3 ± 8.2	76.0 ± 12.4	.978
NYHA functional class III/IV [*n* (%)]	5 (17.9)	5 (23.8)	21 (77.8)	<.001
White blood cell count (10^9^/l)	5.3, 1.1	5.1, 1.6	6.6, 3.2	.016
Lymphocyte count (10^9^/l)	1.8, 0.9	1.6, 0.7	1.8, 0.7	.715
Haemoglobin (g/l)	134.8 ± 15.2	132.1 ± 12.9	137.3 ± 18.3	.536
Red blood cell distribution width (%)	12.6, 1.2	12.5, 1.3	13.6, 2.1	.009
Aspartate aminotransferase (U/l)	21.0, 28.0	17.0, 14.5	24.0, 33.0	.311
Creatinine (μmol/l)	59.0, 22.3	59.0, 21.0	63.0, 26.0	.788
Uric acid (μmol/l)	345.4 ± 110.0	323.7 ± 78.4	389.4 ± 88.9	.052
Fasting blood glucose (mmol/l)	4.7, 0.8	5.0, 1.0	5.2, 1.1	.038
Partial thromboplastin time (s)	28.7, 3.7	28.5, 3.3	28.6,4.6	.918
Prothrombin time (s)	11.5, 1.0	11.2, 1.2	12.0, 2.0	.004
D-Dimer (μg/ml)	0.2, 0.4	0.3, 0.3	0.5, 0.5	.01
Total cholesterol (mmol/l)	4.2 ± 0.8	4.1 ± 1.0	4.3 ± 0.8	.654
Triglycerides (mmol/l)	1.1, 0.7	1.1, 0.6	1.1, 0.5	.887
HDL-C (mmol/l)	1.1, 0.5	1.0, 0.6	1.0, 0.3	.604
LDL-C (mmol/l)	2.6 ± 0.6	2.5 ± 0.6	2.8 ± 0.6	.204
NT-proBNP (pg/ml)	62.1, 74.5	131.7, 152.3	677.9, 1353.0	<.001
Left atrial diameter (mm)	34.0, 6.5	37.0, 13.0	42.0, 15.0	<.001
Left ventricular end-systolic diameter (mm)	29.5, 5.0	28.0, 5.0	31.0, 9.0	.203
Left ventricular end-diastolic diameter (mm)	46.0, 7.8	44.0, 9.0	47.0, 13.0	.389
Peripheral oxygen pressure (mm Hg)	95.5, 8.7	93.0, 8.0	75.4, 16.1	<.001
Pulmonary artery systolic pressure (mm Hg)	28.0, 7.8	43.0, 20.0	72.0, 27.0	<.001
Mean pulmonary artery pressure (mm Hg)	21.0, 4.8	28.0, 4.5	44.0, 14.0	<.001
Pulmonary vascular resistance (wood)	3.4, 2.0	3.6, 2.3	7.6, 5.6	<.001
Mixed venous oxygen saturation (%)	77.6, 8.0	80.8, 11.6	76.5, 16.0	.172
Pulmonary circulation blood volume ratio	1.2, 0.7	1.3, 1.5	1.6, 1.7	.178

Gender is represented by corresponding numbers or percentages, other data are represented as mean ± standard deviation or median and inter-quartile range (IQR).

BMI, body mass index; HDL-C, high-density lipoprotein cholesterol; LDL-C, low-density lipoprotein cholesterol; PAH, pulmonary arterial hypertension.

### Comparison of plasma LCN2 and miR-8078 levels among groups

**Figure 1:**
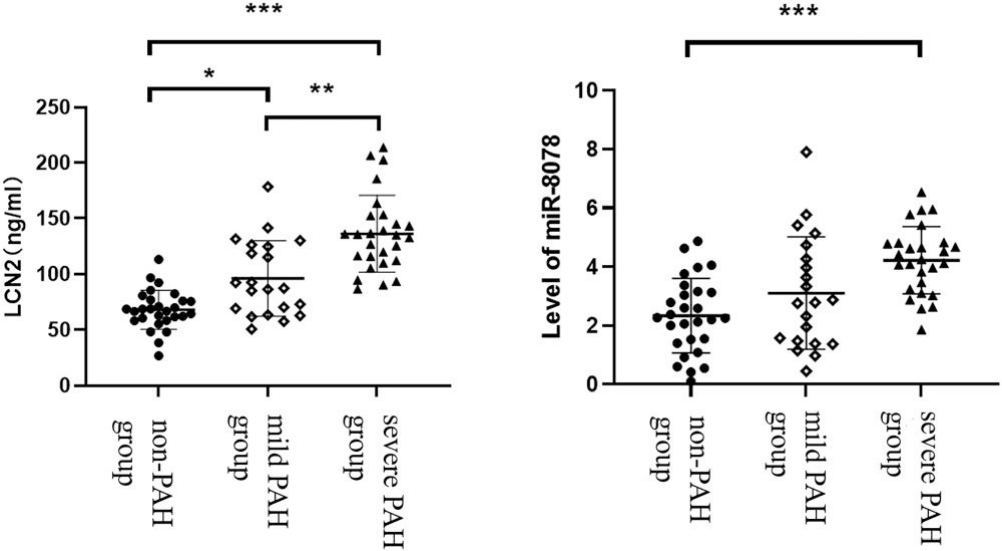
Analysis of the LCN2 levels among the different groups (*P < .05; **P < .01)

**Figure 2:**
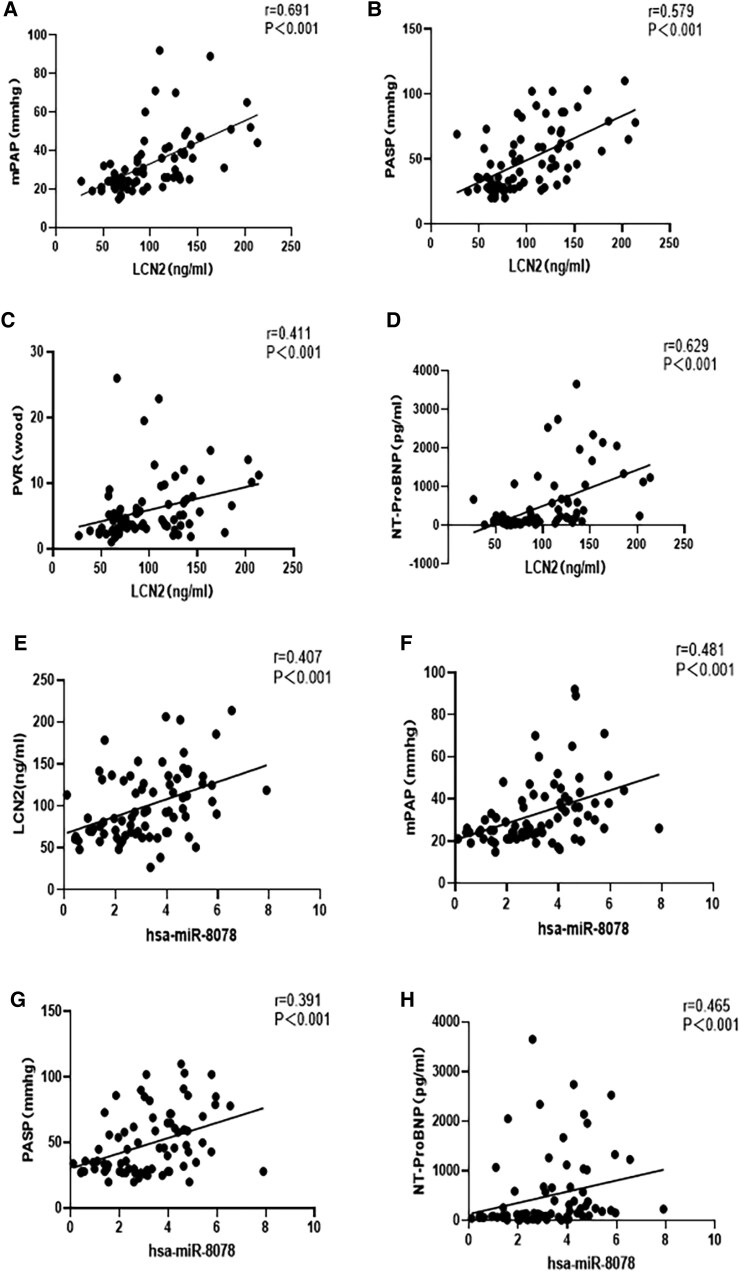
(*A*) Positive correlation between LCN2 and mPAP; (*B*) positive correlation between LCN2 and PASP; (*C*) positive correlation between LCN2 and PVR; (*D*) positive correlation between LCN2 and NT-proBNP; (*E*) positive correlation between miR-8078 and LCN2; (*F*) positive correlation between miR-8078 and mPAP; (*G*) positive correlation between miR-8078 and PASP; and (*H*) positive correlation between miR-8078 and NT-proBNP

The LCN2 expression was much higher in the moderate-to-severe PAH group than in the non-PAH and mild PAH groups. However, LCN2 was up-regulated in the mild PAH group when compared with the non-PAH group. Moreover, the miR-8078 expression was enhanced in the moderate-to-severe PAH group when compared with the non-PAH group (*P* < .05), while no clear differences in terms of miR-8078 were found among the other groups (*Table 2* and *Figures 1 and 2*).

**Table 2:** Levels of LCN2 and miR-8078 among the different groups

Parameters	No PAH group (*n* = 28)	Mild PAH group (*n* = 21)	Moderate-severe PAH group (*n* = 27)
miR-8078	2.3 ± 1.3	3.1 ± 1.9	4.2 ± 1.1^a^
LCN2 (ng/ml)	67.7, 17.7	87.3, 59.2^a^	135.1, 40.2^a,b^

miR-8078 follows a normal distribution and is represented as mean ± standard deviation; LCN2 does not follow a normal distribution and is represented as median and inter-quartile range.

LCN2, Lipocalin 2; PAH, pulmonary arterial hypertension.

^a^Compared with no PAH group.

^b^Compared with mild PAH group.

### Correlation analysis

LCN2 showed significant correlations with the patients’ gender, age, white blood cell count, red blood cell distribution width, uric acid, peripheral oxygen pressure, NYHA functional class, NT-proBNP, PASP, mPAP, PVR, and ratio of pulmonary to systemic blood flow (QP/QS) (where the correlation coefficients were 0.264, 0.337, 0.255, 0.257, 0.269, −0.422, 0.372, 0.629, 0.579, 0.691, 0.411, and 0.315, respectively; *P* < .05). Furthermore, miR-8078 was associated with the patients’ age, red blood cell distribution width, uric acid, peripheral oxygen pressure, NYHA functional class, NT-proBNP, PASP, and mPAP (where the correlation coefficients were 0.294, 0.289, 0.235, −0.368, 0.322, 0.465, 0.391, and 0.481, respectively; *P* < .05) (*Table 3* and *Figure 2A–H*).

**Table 3:** Correlation of LCN2 and miR-8078 with the variables

Parameters	LCN2		miR-8078	
	*r*-value	*P*-value	*r*-value	*P*-value
Female	0.264	.021*	0.037	.753
Age (years)	0.337	.003**	0.294	.010*
White blood cell count	0.255	.026*	0.162	.162
Haemoglobin (g/l)	−0.047	.689	−0.059	.615
Red blood cell distribution width (%)	0.257	.025*	0.289	.011*
Uric acid (μmol/l)	0.269	.019*	0.235	.041*
Fasting blood glucose (mmol/l)	0.081	.488	0.169	.144
Peripheral oxygen pressure (mm Hg)	−0.422	<.001	−0.368	<.01**
NYHA functional classification III/I	0.372	<.01**	0.322	.005**
NT-proBNP (pg/ml)	0.629	<.001	0.465	<.001
Pulmonary artery systolic pressure (mm Hg)	0.579	<.001	0.391	<.001
Mean pulmonary artery pressure (mm Hg)	0.691	<.001	0.481	<.001
Pulmonary vascular resistance (wood)	0.411	<.001	0.201	.082
Pulmonary artery oxygen saturation (%)	0.064	.582	0.026	.823
Ratio of pulmonary circulation blood volume	0.315	.006**	0.201	.082

^*^ *P* < .05; **P < .01.

LCN2, lipocalin 2.

### ROC curve

**Figure 3:**
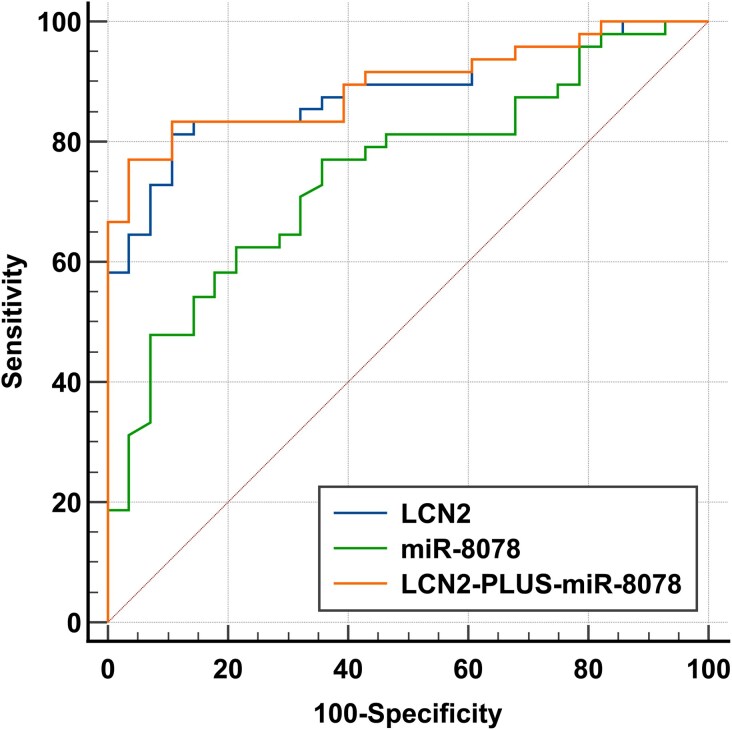
ROC curves of the plasma LCN2, miR-8078, and combined diagnosis of PAH in patients with CHD

In estimating the potential of LCN2 and miR-8078 in the early diagnosis of CHD-PAH, the ROC curve analysis revealed that when the threshold for miR-8078 was set at 2.6, its sensitivity and specificity for the diagnosis of PAH were 77.1% and 64.3%, respectively. Moreover, when the threshold for LCN2 was set at 85.5 ng/ml, its sensitivity for the diagnosis of PAH was 81.3%, with a specificity of 89.3%. The combination of the two markers for the diagnosis of PAH only exhibited a numerical increase AUC and specificity scores (*Table 4* and *Figure 3*).

**Table 4:** ROC curves of the plasma LCN2, miR-8078, and combined diagnosis of PAH in patients with CHD

Parameters	AUC	Youden index	Sensitivity (%)	Specificity (%)	*P*-value
LCN2	0.883 (0.789–0.946)	0.7054	81.3 (67.4–91.1)	89.3 (71.8–97.7)	<.0001
miR-8078	0.749 (0.636–0.841)	0.4137	77.1 (62.7–88.0)	64.3 (44.1–81.4)	<.0001
LCN2 + miR-8078	0.896 (0.804–0.954)	0.7351	77.1 (62.7–88.0)	96.43 (81.7–99.9)	<.0001

LCN2, Lipocalin 2; AUC, area under the curve.

### Univariate and multi-variate logistic regression analysis

The univariate logistic regression indicated that the patients’ gender, age, left atrial diameter, peripheral oxygen pressure, NYHA functional class, NT-proBNP, LCN2, and miR-8078 were all risk factors for CHD-PAH. Therefore, a multi-variate logistic regression was performed to further estimate the role of these variables. As shown in *Table 5*, the plasma LCN2 was identified as an independent risk factor for CHD-PAH (odds ratio [OR] = 1.061; 95% confidence interval [CI], 1.024–1.100; *P* < .05). However, miR-8078 was not independently associated with PAH in this model (OR = 1.483; 95% CI, 0.862–2.551; *P* = .155).

**Table 5:** Univariate and multi-variate logistic regression analysis of patients with CHD-PAH

Parameters	Univariate analysis		Multi-variate analysis	
	OR (95% CI)	*P*-value	OR (95% CI)	*P*-value
Female	2.915 (1.070–7.943)	.036	3.490 (0.676–18.012)	.136
Age (years)	1.041 (1.008–1.074)	.013	0.975 (0.919–1.034)	.396
BMI (kg/m²)	0.985 (0.871–1.113)	.805		
Left atrial diameter (mm)	1.099 (1.027–1.176)	.006	1.094 (0.967–1.238)	.155
Left ventricular end-systolic diameter (mm)	1.035 (0.955–1.122)	.401		
Left ventricular end-diastolic diameter (mm)	1.024 (0.956–1.097)	.497		
White blood cell count (10^9^/l)	1.252 (0.969–1.618)	.086		
Lymphocyte count (10^9^/l)	0.801 (0.376–1.704)	.564		
Red blood cell distribution width (%)	1.525 (0.933–2.491)	.092		
Haemoglobin (g/l)	1.001 (0.971–1.031)	.962		
Aspartate aminotransferase (U/l)	0.994 (0.970–1.018)	.615		
Creatinine (μmol/l)	1.012 (0.983–1.042)	.413		
Uric acid (μmol/l)	1.002 (0.997–1.007)	.507		
D-Dimer (μg/ml)	1.499 (0.726–3.098)	.274		
Total cholesterol (mmol/l)	0.970 (0.560–1.681)	.914		
Triglycerides (mmol/l)	0.754 (0.441–1.290)	.302		
Peripheral oxygen pressure (mm Hg)	0.922 (0.877–0.970)	.002	0.939 (0.854–1.032)	.192
Mixed venous oxygen saturation (%)	1.002 (0.945–1.062)	.951		
NYHA functional class III/IV	5.436 (1.771–16.686)	.003	0.604 (0.056–6.577)	.679
NT-proBNP (pg/ml)	1.004 (1.001–1.008)	.013	1.000 (0.997–1.004)	.819
LCN2 (ng/ml)	1.068 (1.035–1.101)	<.001	1.061 (1.024–1.100)	.001
miR-8078	1.913 (1.312–2.789)	.001	1.483 (0.862–2.551)	.155

LCN2, lipocalin 2; PAH, pulmonary arterial hypertension.

## Discussion

A key objective of this study was to explore whether combining LCN2 and miR-8078 could improve diagnostic accuracy for CHD-PAH. Our multi-variable analysis revealed that while both markers were elevated in patients with PAH, only LCN2 emerged as a robust, independent predictor. The addition of miR-8078 to the model did not result in a statistically significant improvement in the AUC, suggesting that its diagnostic contribution may largely overlap with that of LCN2 in this cohort.

In this study, the plasma LCN2 expression was found to be significantly increased in the moderate-to-severe PAH group when compared with the other groups. All the enrolled CHD patients had left-to-right shunts, which was identified as a crucial trait of CHD-PAH. Oxidative stress stimulation can provoke PAEC damage and release of inflammatory factors, thereby leading to inflammatory cell infiltration.^6,14^ The elevation of the plasma LCN2 levels in the CHD-PAH patients included in this study might be attributed to this issue. Hemodynamics represents a key index used to evaluate the progression of PAH. The correlation analysis revealed the positive association of LCN2 with the patients’ mPAP, PASP, PVR, and QP/QS, which accorded with the findings of previous research.^4^ LCN2 was determined to be up-regulated as the PAH severity increased, indicating that LCN2 can reflect the mPAP level and contribute to the evaluation of CHD-PAH severity. In contrast, Takahashi et al. found that the serum LCN2 level was negatively correlated with the right ventricular systolic pressure in patients with systemic sclerosis-associated PAH and normal renal function.^5^ Various aetiologies and mechanisms of pulmonary hypertension might be the cause of this inconsistency. Moreover, NT-proBNP is related to diverse hemodynamic parameters correlated with survival in cases of PAH,^15^ and it is currently the only biomarker recognized by guidelines for the assessment of the prognosis in PAH.^16^ In this study, LCN2 showed a significant positive correlation with NT-proBNP, further indicating the potential of the former in evaluating the severity and prognosis of CHD-PAH. Hypoxia exposure induces inflammatory reactions in the vascular wall, thereby significantly contributing to both structural remodelling and sustained vasoconstriction in the pulmonary circulation, which suggests chronic hypoxia to be a promoter of PAH development.^17^ The present study demonstrated a significant negative correlation between LCN2 and the peripheral oxygen partial pressure, indicating that LCN2 could reflect the oxygen saturation index of patients, and therefore, allow for a timely response to the disease. Interestingly, LCN2 was significantly positively related to the NYHA functional classification and served as an indicator for evaluating the right heart function in patients with CHD-PAH to some extent. According to the ROC curve results, when the threshold for LCN2 was set at 85.5 ng/ml, its sensitivity and specificity for the diagnosis of CHD-PAH were 81.3% and 89.3%, respectively. Identifying both risk factors and predictive factors for CHD-PAH contributes to our understanding of the disease. Based on the binary logistic regression analysis, LCN2 served as an independent risk factor for CHD-PAH. Indeed, every 1 ng/ml increase in LCN2 was associated with a 6.8% increased risk of PAH in CHD patients. Based on the above data, we speculate that LCN2 has diagnostic value in relation to CHD-PAH.

Furthermore, miRNAs are pivotal participants in the pathogenesis of cardiovascular diseases, including PAH. They have also been found to modulate cell proliferation and vascular remodelling. For example, the involvement of miR-21, −20, −145, and −204 in PAH progression has been confirmed.^12,18^ Chen *et al*. preliminarily demonstrated the differential expression of miR-19a, −130a, and −27b in CHD-PAH and their possible association with the systemic inflammatory response and endothelial cell activation in patients with PAH.^19^ Additionally, miR-371b-5p has been reported to be up-regulated and to inhibit PAEC apoptosis through the PI3K/Akt pathway in rats with monocrotaline-induced PAH.^11^ Moreover, the knockdown of miR-1 expression alleviated the right ventricular hypertrophy and fibrosis in an animal model of PAH, which may be associated with the PI3K/AKT pathway.^13^ Similarly, miR-325-3p may alleviate right ventricular fibrosis in rats with PAH by regulating the PI3K/AKT signalling pathway,^20^ while miR-19a may regulate human PASMC proliferation and migration via the PTEN/PI3K/AKT axis.^21^ In addition, in a rat model of dilated cardiomyopathy, miR-132 down-regulated PTEN to activate the PI3K/Akt pathway, thereby restraining both cell apoptosis and cardiac fibrosis.^22^ In summary, it is evident that miRNAs and the PI3K/Akt signalling pathway significantly interact in PAH. It is acknowledged that miR-8078 may be a key mediator of RA development and may affect synovial macrophages,^9^ while the PI3K/AKT pathway is implicated in the pathogenesis of RA. Therefore, we speculate that miR-8078 may play a mediating role in PAH due to the influence of the PI3K/AKT pathway.

This study found that the plasma miR-8078 level was markedly up-regulated in the moderate-to-severe PAH group when compared with the non-PAH group. Moreover, miR-8078 showed a strong positive correlation with the patients’ mPAP, PASP, and NT-proBNP. We hypothesized that miR-8078 regulated the progression of PAH by promoting the proliferation of PASMC, potentially serving as an indicator of CHD-PAH severity. Setting the miR-8078 threshold at 2.6 provided a sensitivity of 77.1% and a specificity of 64.3% with regard to the diagnosis of CHD-PAH. The univariate regression analyses revealed elevated miR-8078 to be a risk factor for CHD-PAH.

A noteworthy finding of our study is that while both LCN2 and miR-8078 were significant predictors in the univariate analysis, only LCN2 remained an independent predictor of moderate-to-severe PAH in the multi-variate model. This underscores the robust nature of LCN2 as a potential biomarker in this setting, suggesting it may play a core role in the disease process. Based on our findings, LCN2 in particular exhibited potential significance in several areas: (i) as a promising biomarker for the diagnosis of CHD-PAH; (ii) for evaluating the severity of CHD-PAH, given its strong correlation with hemodynamic parameters; and (iii) for providing new insights into the pathogenesis of CHD-PAH. The potential roles in predicting prognosis, risk assessment, or guiding treatment are speculative at this stage and would require dedicated longitudinal studies. Since mPAP measured via RHC is the gold standard for PAH diagnosis, and given that LCN2 levels correlate with mPAP, it may serve as a useful non-invasive indicator to help monitor disease status and potentially predict the progression of PAH. This finding does not necessarily diminish the potential biological relevance of miR-8078. It is possible that in a larger cohort, or in a different clinical context, the unique contribution of miR-8078 might become more apparent. Our ROC curve analysis demonstrated that although the combined model produced a numerically higher AUC, the addition of miR-8078 did not significantly enhance the diagnostic performance of LCN2. The correlation analysis in this study indicated a strong positive correlation between LCN2 and miR-8078, suggesting a potential co-regulatory relationship or shared upstream pathway in the context of pulmonary vascular remodelling in CHD. Further investigation, perhaps with larger sample sizes and animal models, is needed to fully elucidate the distinct role of miR-8078.

Several limitations of this study should be acknowledged. First, the study is constrained by a relatively small sample size from a single centre, which may limit the statistical power and external validity of our findings. Furthermore, the patient cohort consisted predominantly of individuals with non-cyanotic CHD (94.7%), with only a small number of patients with cyanotic CHD. This imbalance precluded a statistically robust subgroup analysis to compare biomarker levels between different CHD types. Although we hypothesize that LCN2 and miR-8078 levels would be higher in cyanotic CHD due to their correlation with more severe hemodynamic stress, this remains to be confirmed. Second, the cross-sectional nature of the study establishes a strong association and diagnostic potential for LCN2 and miR-8078 in CHD-PAH, but it cannot determine causality or prognostic significance. Therefore, future research is essential to build upon these initial findings. Validation in larger, multi-centre prospective cohorts is necessary to confirm the robustness and generalizability of these biomarkers, assess their prognostic value, and include a more balanced representation of both cyanotic and non-cyanotic CHD subtypes. Furthermore, while our study identifies a clinical association, mechanistic studies are required to elucidate the underlying biological pathways. Our preliminary analysis suggests that LCN2 and miR-8078 may exert their influence on PAH pathogenesis via the PI3K/Akt signalling pathway. Investigating this hypothesis through dedicated *in vitro* and *in vivo* experiments will be a central focus of our future research.

## Conclusion

This study confirms that plasma levels of LCN2 and miR-8078 are significantly elevated in patients with CHD-PAH. Our analysis establishes LCN2 as a strong and independent diagnostic biomarker for CHD-PAH, showing a significant positive correlation with hemodynamic parameters of disease severity, such as mPAP and PASP. While miR-8078 levels were also correlated with disease severity in univariate analysis, it did not emerge as an independent predictor in our multi-variable model. Consequently, a combined LCN2 and miR-8078 model, while yielding a numerically higher AUC, did not offer a statistically significant improvement in diagnostic performance over LCN2 alone. The lack of significant improvement in the combined model may be attributed to the strong correlation between these two markers. Therefore, our findings support the use of LCN2 as a valuable, non-invasive biomarker to aid in the diagnosis and severity assessment of CHD-PAH. The role of miR-8078, while biologically interesting, requires further investigation in larger cohorts to determine if it has a distinct or complementary role in the complex pathogenesis of this disease.

## Data Availability

The data underlying this article will be shared on reasonable request to the corresponding author.

## Pre-registered Clinical Trial Number

None supplied.
